# Reduced Immunity Regulator MAVS Contributes to Non-Hypertrophic Cardiac Dysfunction by Disturbing Energy Metabolism and Mitochondrial Homeostasis

**DOI:** 10.3389/fimmu.2022.919038

**Published:** 2022-07-01

**Authors:** Qian Wang, Zhenzhen Sun, Shihan Cao, Xiuli Lin, Mengying Wu, Yuanyuan Li, Jie Yin, Wei Zhou, Songming Huang, Aihua Zhang, Yue Zhang, Weiwei Xia, Zhanjun Jia

**Affiliations:** ^1^ Nanjing Key Laboratory of Pediatrics, Children’s Hospital of Nanjing Medical University, Nanjing, China; ^2^ Jiangsu Key Laboratory of Pediatrics, Nanjing Medical University, Nanjing, China; ^3^ Department of Nephrology, Children’s Hospital of Nanjing Medical University, Nanjing, China

**Keywords:** MAVS, cardiac dysfunction, mitochondrial dysfunction, energy metabolism, oxidative stress

## Abstract

Cardiac dysfunction is manifested as decline of cardiac systolic function, and multiple cardiovascular diseases (CVDs) can develop cardiac insufficiency. Mitochondrial antiviral signaling (MAVS) is known as an innate immune regulator involved in viral infectious diseases and autoimmune diseases, whereas its role in the heart remains obscure. The alteration of MAVS was analyzed in animal models with non-hypertrophic and hypertrophic cardiac dysfunction. Then, MAVS-deficient mice were generated to examine the heart function, mitochondrial status and energy metabolism. *In vitro*, CRISPR/Cas9-based gene editing was used to delete MAVS in H9C2 cell lines and the phenotypes of mitochondria and energy metabolism were evaluated. Here we observed reduced MAVS expression in cardiac tissue from several non-hypertrophic cardiac dysfunction models, contrasting to the enhanced MAVS in hypertrophic heart. Furthermore, we examined the heart function in mice with partial or total MAVS deficiency and found spontaneously developed cardiac pump dysfunction and cardiac dilation as assessed by echocardiography parameters. Metabonomic results suggested MAVS deletion probably promoted cardiac dysfunction by disturbing energy metabolism, especially lipid metabolism. Disordered and mitochondrial homeostasis induced by mitochondrial oxidative stress and mitophagy impairment also advanced the progression of cardiac dysfunction of mice without MAVS. Knockout of MAVS using CRISPR/Cas9 in cardiomyocytes damaged mitochondrial structure and function, as well as increased mitochondrial ROS production. Therefore, reduced MAVS contributed to the pathogenesis of non-hypertrophic cardiac dysfunction, which reveals a link between a key regulator of immunity (MAVS) and heart function.

## Introduction

Cardiac dysfunction is a common advanced feature of various CVDs such as myocardial infarction, myocarditis, and hypertension (1, 2). Data from WHO indicate CVD is the most common cause of death in the world and in particularly ischemia heart disease and stroke are the top two killers, contributing to 16% and 11% of the world’s death. Since the global burden of CVDs is huge, prevent and delay the progression of CVDs and protect cardiac function are of great importance.

Mitochondria, the powerhouses of cells, are especially vital to cells with high energy requirements, such as cardiomyocytes. Recent reviews have summarized several mitochondria-mediated mechanisms including a bottleneck of metabolic flux, iron imbalance, oxidative stress, inflammation, mitochondrial dynamics disorder, and impaired mitophagy that promote cardiac dysfunction (3, 4). Therefore, considering the importance of mitochondria in maintaining normal cardiac contraction and relaxation, targeting mitochondria may improve heart’s function. Indeed, some studies have demonstrated that the protection or restoration of mitochondrial function can improve cardiac function (5–8).

MAVS, a protein mainly located in the outer membrane of the mitochondria (OMM), is well known for its key role in antiviral response (9). MAVS was recently found to participate in other diseases, such as systemic lupus erythematosus (SLE). MAVS oligomerization was detected in plasma and peripheral blood monocytes (PBMCs) from patients with SLE, which induced endogenous prion-like MAVS aggregates (10). Moreover, MAVS-mediated nuclear factor kappa B (NF-κB) activation also promoted neuroinflammation in experimental autoimmune encephalomyelitis (EAE) and multiple sclerosis (MS) (11). However, few studies have documented the role of MAVS in cardiac function and CVDs. As immune regulator, spontaneous MAVS signaling activation independent of RIG-1 and MDA5 is indispensable for high basal IFNβ level in unstimulated cardiac myocytes, serving as reserve army in case of virus attack (12). Recently, it was reported that MAVS acted as downstream of nucleotide-binding oligomerization domain-containing protein 1/receptor-interacting protein 2 (NOD1/RIP2) to promote cardiac hypertrophy following pressure overload induced by transverse aortic constriction (TAC) (13). Here we found that a partial or complete MAVS deficiency resulted in reduction of the cardiac function and enlarged hearts in mice. Mechanistically, MAVS deficiency disrupts mitochondrial function, energy production, and lipid metabolism. Our findings suggest that MAVS may serve as a potential target for myocardial protection.

## Materials and Methods

### Animals

MAVS gene knockout (MAVS^-/-^) mice with a C57BL/6N background were raised under specific pathogen-free conditions. Mouse tails were acquired for genotyping when newborn mice reached 7 days. The following primers were used for genotyping: F1: 5′-TAGCTGTGAGGCAGGACAGGTAAGG-3′; F2:5′-AGCCAAGATTCT AGAAGCTGAGAA-3′; F3: 5′-GTGGAATGTGTGCGAGGCCAGAGGC-3′. The animals with polymerase chain reaction (PCR) products of 350 bp in length were considered MAVS^-/-^ mice. The animals with PCR products of 250 bp in length were considered wild-type (WT) mice. In this study, mice aged 2-3, 12-16, 24, and 48 weeks were used. All mice were fed a standard laboratory diet and maintained under a 12:12-h light-dark cycle. This study was approved by the Institutional Animal Care and Use Committee of Nanjing Medical University. All animal work was performed at Animal Research Center of Nanjing Medical University (Nanjing, China).

### Echocardiography

Cardiac structural and functional parameters were assessed using echocardiography (Visualsonics VEVO 2100, Toronto, Canada). The mice were anesthetized by inhalation of isofluorane and the chest was shaved. Two-dimensional echocardiography was performed to collect short-axis M-mode images. The ejection fraction (EF), fractional shortening (FS), chamber volume, internal diameter at the end of systole and diastole, and mass of the left ventricle were recorded and analyzed under blinding condition.

### Sample Preparation for Metabolomics Assay and Liquid Chromatography Dual Mass Spectrometry Analysis

The heart tissues were flash frozen in liquid nitrogen, weighed (80 mg), then placed in 200 μL of purified water and vortexed for 60 s. The samples were then extracted with 800 μL of methanol acetonitrile (1:1, v/v), vortexed, ultrasonicated, and concentrated. The supernatants were collected, frozen, dried, and stored at -80°C. The samples were then separated using an Agilent 1290 Infinity LC and analyzed by AB Triple TOF 5600/6600.

### Metabolite Identification and Multivariate Data Analysis

Peak areas were extracted from the raw data to generate a data matrix. After normalizing of the peak areas of metabolites to the protein concentrations, principal component analysis was performed. Student’s t-test was subsequently conducted on the acquired metabolomics data, and a significance level of P< 0.05. Molecular identification of the significantly changed metabolites was achieved by matching the masses and secondary spectrum against the metabolome databases. Metabolic pathway enrichment analysis was performed using pathway enrichment analysis.

### CRISPR/Cas9

The CRISPR/Cas9 protocol was described in a previous study. Briefly, sgRNAs targeting rat MAVS were designed from a web interface of CRISPR design (http://crispr.mit.edu/). The oligonucleotides were synthesized by Genebay Biotech (Nanjing, China) and the sequences were listed in **Table 1**. The oligonucleotides were then annealed into double chains after being phosphorylated and cloned into the pSpCas9(BB)-2A-Puro (PX459) vector. The sequenced CRISPR/Cas9 plasmids targeting MAVS were transfected into H9C2 cells using Lipofectamine^®^ 2000, and puromycin (4 µg/mL) was used to screen for positive cells. After three days of screening, live cells were seeded in a 96-well plate for monoclonal expansion. An empty vector was used as a negative control.

**Table 1:** Primer Sequences.

Gene name	Forward primer sequence (5′-3′	Reverse primer sequence (5′-3′)
Mouse MAVS	CTGCCTCACAGCTAGTGACC	CCGGCGCTGGAGATTATTG
Mouse ACC1	CTTCCTGACAAACGAGTCTGG	CTGCCGAAACATCTCTGGGA
MouseACC2	CGCTCACCAACAGTAAGGTGG	GCTTGGCAGGGAGTTCCTC
Mouse CD36	ATGGGCTGTGATCGGAACTG	GTCTTCCCAATAAGCATGTCTCC
Mouse CPT1α	CCTGTCGAAACATCTACCAT	AAGTGTCGGCAGACCTATT
Mouse PPARα	AGAGCCCCATCTGTCCTCTC	ACTGGTAGTCTGCAAAACCAAA
Mouse MCAD	TAACATACTCGTCACCCTTC	ATGCCTGTGATTCTTGCT
Mouse mt-ATP6	CCATAAATCTAAGTATAGCCATTCCAC	AGCTTTTTAGTTTGTGTCGGAAG
Mouse mt-ATP8	ACATTCCCACTGGCACC	GGGGTAATGAATGAGGC
Mouse mt-ND4L	GCCATCTACCTTCTTCA	TAGGGCTAGTCCTACAGC
Mouse mt-COX1	CAGACCGCAACCTAAACACA	TTCTGGGTGCCCAAAGAAT
Mouse mt-COX2	GCCGACTAAATCAAGCAACA	CAATGGGCATAAAGCTATGG
Mouse mt-COX3	CGTGAAGGAACCTACCAAGG	ATTCCTGTTGGAGGTCAGCA
Mouse mt-ND1	ACACTTATTACAACCCAAGAACACAT	TCATATTATGGCTATGGGTCAGG
Mouse mt-ND2	CCATCAACTCAATCTCACTTCTATG	GAATCCTGTTAGTGGTGGAAGG
Mouse mt-ND3	CCCCAAATAAATCTGTA	CTCATGGTAGTGGAAGT
Mouse mt-ND4	GCTTACGCCAAACAGAT	TAGGCAGAATAGGAGTGAT
Mouse mt-ND5	GCCAACAACATATTTCAACTTTTC	ACCATCATCCAATTAGTAGAAAGGA
Mouse mt-ND6	GGGAGATTGGTTGATGTA	ATACCCGCAAACAAAGAT
Mouse mt-Cytb	GAGGTTGGTTCGGTTTTGG	GTTTTGAAAGGGTGGGTGAC
Mouse GAPDH	AGGTCGGTGTGAACGGATTTG	TGTAGACCATGTAGTTGAGGTCA
Rat mt-ND1	GTTGCCCAAACCATCTCTTACG	TGAAGAATAGGGCGAATGGTCC
Rat mt-ND2	TACCCGAAGTCACCCAAGGA	AGGCGCCAACAAAGACTGAT
Rat mt-ND3	CCATATGAATGTGGCTTCGACC	TGGTTGTTTGAATCGCTCATGG
Rat mt-ND4	ACCTAGCACTACCACCCCTAAT	GGTTGGAGGTTGTTTATGTGGC
Rat mt-ND4L	TCTACTCTCCTCTGCCTAGAAGG	AGGCTAAACCTACTGCTGCTTC
Rat mt-ND5	ACACTCTGATCCCCACATTAACC	TTCCTGTGGCTGCAATTAGTAGT
Rat mt-ND6	GGTGGGCTTGGATTGATTGTTAG	CCAATTAGACCCTCAAGTCTCCG
Rat mt-Cytb	TCCCTTACATTGGGACTACCCT	TGGGAATGGAGCGTAGAATAGC
Rat mt-COX1	AATGACACATGAGCAAAAGCCC	TCTTCGAATGTGTGGTAGGGTG
Rat mt-COX2	GCTTACCCATTTCAACTTGGCTT	CGTCTATTGTGCTTGTGTGTGTT
Rat mt-COX3	GCTCTTCTACTTACATCCGGCTT	CTTCTATTAGGCTGTGATGGGCT
Rat mt-ATP6	AATCAGCAACCGACTACACTCA	AGAAGCCCTAGAAGGTTGGTTG
Rat mt-ATP8	ATGCCACAACTAGACACATCCA	TATAGTTTTGGGGGAGGGAGGT
Rat GAPDH	TCTCTGCTCCTCCCTGTTC	ACACCGACCTTCACCATCT
Rat FASN	ATCCTAGGCATCCGCGACCT	TCACGAATGGGTAGCACCAGAT
Rat ACC1	GCCATCCGGTTTGTTGTCA	GGATACCTGCAGTTTGAGCCA
Rat ACC2	AAGTCATCTCCTGCTTTGCC	TGCAAACTCATCTCTCGCTCT
Rat CPT1α	CTGCTGTATCGTCGCACATTAG	GTTGGATGGTGTCTGTCTCTTCC
Mouse MAVS sgRNA-1: GTTGTAGCGGATATACTTAT		
Mouse MAVS sgRNA-2: AGGAAACCAGGGCACACTCT		

### Histology

After animal sacrifice, the heart tissues were fixed immediately in 4% paraformaldehyde overnight or immediately frozen in optimal cutting temperature (OCT) compound. Hematoxylin-eosin (HE) staining was performed of 3 µm thick cross-sectional slices cut from paraffin-embedded tissues following the manufacture’ instructions. Frozen heart sections (7 μm) were prepared for Oil red O (ORO) staining using a modified Oil Red O Staining Kit (C0158S, Beyotime Biotech, Nantong, China).

### Measurements of Serum Creatine Kinase-MB and Lactate Dehydrogenase

Blood taken from the inferior caval vein was centrifuged at 3000 rcf for 20 min and the serum was collected. A serum biochemical autoanalyzer was used to measure the levels of serum creatine kinase-MB (CK-MB) and lactate dehydrogenase (LDH).

### Cell Culture and Treatment

The rat heart-derived H9C2 cardiac cells were purchased from American Type Culture Collection (ATCC) and cultured in Dulbecco’s modified Eagle’s medium (DMEM) with 10% fetal bovine serum (FBS), 1% penicillin, and streptomycin in an atmosphere of 5% CO2 at 37°C. When the cells reached 90% confluence, they were harvested for appropriate analysis.

### Measurement of Mitochondrial Membrane Potential and Mitochondrial Reactive Oxygen Species Production

The mitochondrial membrane potential and reactive oxygen species production of H9C2 were detected using tetramethylrhodamine, methyl ester (TMRM; I34361; Thermo Fisher Scientific, Waltham, MA, USA), Mito-Tracker Green (C1048; Beyotome, Shanghai) and MitoSOX™ Red mitochondrial superoxide indicator (M36008; Thermo fisher, Scientific), respectively, according to the manufacturer’s instructions. Briefly, naïve and MAVS^-/-^ H9C2 cells were seeded in a 12-well plate or confocal dish. When they reached to 90% confluence, the cells were incubated with TMRM and Mito-Tracker Green for 30 min or MitoSOX™ for 10 min in the dark at 37°C and washed with phosphate buffered saline three times. Flow cytometry was used to detect fluorescence intensity, and the results were analyzed using Cytexpert software. A laser scanning confocal microscope (CarlZeiss LSM710, Germany) was used to capture the fluorescence images.

### Measurement of ATP Production

An enhanced ATP assay kit (S0027; Beyotime Biotech) was used to measure the ATP content in H9C2 cells using a luciferase reporter assay system (Promega Corporation). Total ATP levels was calculated by normalization to the protein concentrations.

### Transmission Electron Microscopy

To evaluate the morphological changes in myocardial fibers and mitochondria, heart tissues were collected and fixed in 2.5% glutaraldehyde/0.1 M phosphate buffer. Next, the samples were post fixed in 1% OsO4/0.1 M phosphate buffer for 2 h. Ultrathin sections (60 nm) were cut on a microtome, placed on copper grids, stained with uranyl acetate and lead citrate, and examined under an electron microscope (JEM-1010, JEOL Tokyo, Japan). The visual fields of the blinded samples were randomly checked.

### RNA Extraction and Quantitative Reverse Transcription-Polymerase Chain Reaction

Total RNA from heart tissues and H9C2 cells was extracted using TRIzol reagent (Invitrogen, Carlsbad, CA, USA). RNA concentrations were determined using a NanoDrop One spectrophotometer (Thermo Fisher Scientific). Then, the first-strand cDNA was reverse transcribed from 1μg RNA using PrimeScript RT Reagent Kit (TaKaRa, Tokyo, Japan). Quantitative reverse transcription polymerase chain reaction (RT-qPCR) was performed using a SYBR Green Premix Kit (Vazyme, Nanjing, China) in a Roche LightCycler 96 system (Roche, Switzerland). The primer sequences are listed in **Table 1**. GAPDH was used as an internal control.

### Western Blotting

Heart tissues and cells were lysed with RIPA buffer (Beyotime Biotech, Nantong, China) supplemented with a 1:100 proteinase inhibitor for 30 min on ice (Roche, Basel, Switzerland). The samples were centrifuged at 12,000 rcf for 15 min to obtain the supernatant, and the concentrations were measured using the BCA Protein Assay Kit (P0012; Beyotime Biotech). The protein samples (30 μg) were loaded and separated by 10% SDS-PAGE gels, followed by transfer onto polyvinylidene difluoride (PVDF) membranes. The PVDF membrane was blocked with 5% non-fat milk at room temperature for 1 h and then probed with primary antibodies against: MAVS (4983, CST), ND3 (sc-26760, Santa Cruz Biotechnology), ND1(19703-1-AP, Proteintech), Cytochrome C (66264-1-Ig, Proteintech), PARKIN (14060-1-AP, Proteintech), PINK1 (23274-1-AP, Proteintech), P62 (PM045, MBL), LC3 (12741, CST), GAPDH (60004-1-Ig, Proteintech), and β-actin (66009-1-Ig, Proteintech) at 4°C overnight. After incubation with peroxidase-conjugated goat anti-rabbit secondary antibody (A0208, Beyotime) or peroxidase-conjugated goat anti-mouse secondary antibody (A0216, Beyotime) for 1 h at temperature, the expression levels of target proteins were detected using an enhanced chemiluminescence detection system (Bio-Rad, Hercules, CA, US).

### Statistical Analysis

The statistical analysis was performed using GraphPad Prism software (version 8.0; GraphPad Software, La Jolla, CA, USA). All values are presented as mean ± SEM. The statistical analysis was performed using Student’s t-test. Differences were considered significant at values of P < 0.05.

## Results

### Differential Regulation of MAVS in Animal Models With Non-Hypertrophic and Hypertrophic Cardiac Dysfunction

**Figure 1:**
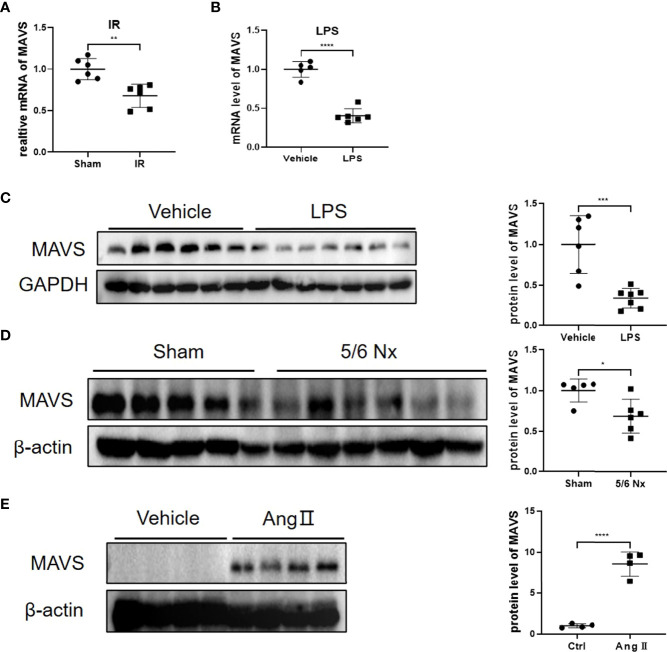
Differential regulation of MAVS in animal models with non-hypertrophic and hypertrophic cardiac dysfunction. **(A)**. The data from GSE4105 illustrated that the MAVS expression (gene ID: 311430) was lower in rats challenged with cardiac ischemia-reperfusion injury (P=0.0019). **(B)**. The mRNA levels of MAVS in heart tissues of mice treated with lipopolysaccharide (LPS, 10mg/kg, 12 h) were significantly downregulated. **(C)**. Representative western blots of MAVS in LPS-treated hearts. Densitometry analysis of the western blots of MAVS. **(D)**. Representative western blots of MAVS in the hearts of mice subjected to 5/6Nx. Densitometry analysis of the western blots of MAVS. **(E)**. Representative western blots of MAVS in Angiotensin II (Ang II)-challenged mice hearts (Ang II, 1.4 mg/kg/day, 4 weeks). Densitometry analysis of the western blots of MAVS. The quantitative results are shown as means ± standard error of the mean (SEM) (*P < 0.05, **P < 0.01, ***P < 0.001 and ****P < 0.0001).

GEO data from GSE4105 demonstrated the significantly reduced mRNA level of MAVS in rats suffering from ischemia-reperfusion (IR) injury (**Figure 1A**). Similarly, we observed reduced expression of MAVS in the hearts of LPS-treated mice at the mRNA and protein levels (**Figures 1B, C**). Besides, MAVS was also downregulated in the injured hearts associated with the advanced chronic kidney disease caused by 5/6 nephrectomy (Nx) (**Figure 1D**). However, angiotensin (Ang) II treatment induced an increased expression of MAVS in hypertrophic heart, which was in agreement with a previous study indicating enhanced MAVS expression in hypertrophic hearts of TAC-mice (**Figure 1E**) (13). The discrepant expression of MAVS suggests MAVS may play different roles in non-hypertrophic and hypertrophic cardiac dysfunction.

### MAVS Deficiency Contributed to Cardiac Pump Dysfunction and Left Ventricular Dilation in Mice

**Figure 2:**
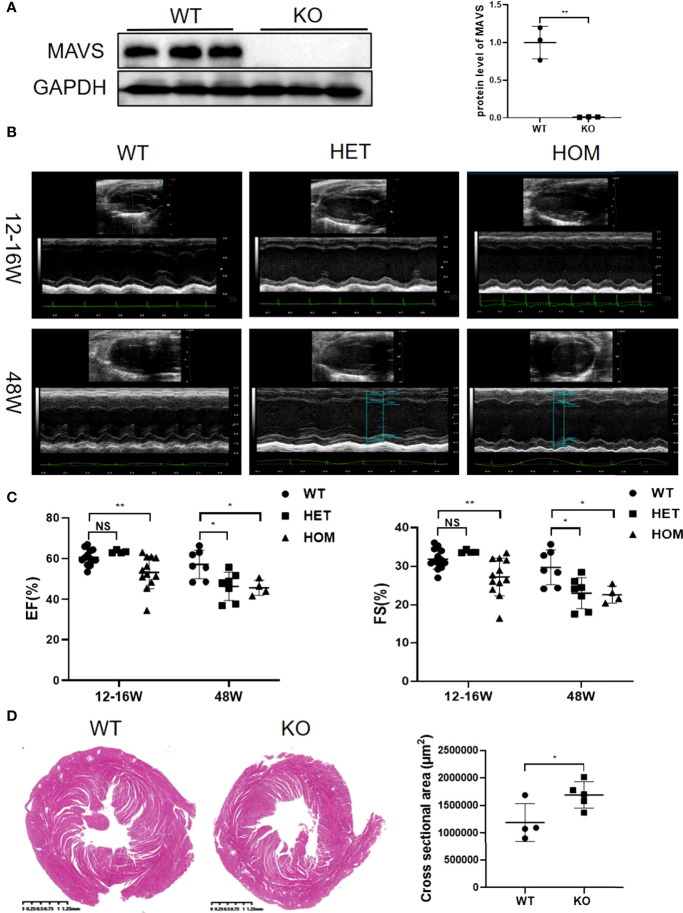
MAVS depletion leads to left ventricular dilation and decreased systolic function in male mice. **(A)**. Representative western blotting analysis of MAVS in the hearts of wild-type (WT) and MAVS^-/-^ mice. Densitometry analysis of the western blots of MAVS. **(B)**. Representative short-axis M-mode echocardiographic images of WT and MAVS^-/-^ mice aged 12-16 weeks and 48 weeks. **(C)**. Ejection fraction and fractional shortening of mice from WT and MAVS^-/-^ groups. **(D)**. Cross-sectional view of the hearts from WT and MAVS^-/-^ mice at the papillary level, (scale bar=0.25mm). Cross-sectional areas of hearts from WT and MAVS^-/-^ mice. The quantitative results are shown as means ± SEM (NS, not significant; *P < 0.05, **P < 0.01).

Inspiring from above results showing the differential regulation of MAVS in multiple cardiac injury models, we next generated MAVS conventional knockout mice (MAVS^-/-^) to investigate the function of MAVS in the heart. Western blotting analysis was used to verify the deletion of MAVS from the hearts of mice (**Figure 2A**). Next, we performed transthoracic echocardiography to evaluate the cardiac function of male WT and MAVS^-/-^ mice. Representative M-mode images of individual mice from WT, heterozygous (Het), and homozygote (Hom) groups of different ages (12-16 weeks and 48 weeks) are shown in **Figure 2B**. Compared with WT mice, MAVS^-/-^ mice displayed reduced cardiac ejection function, as evidenced by a significantly decreased ejection fraction (EF) and fractional shortening (FS) (**Figure 2C**). Interestingly, young Het mice had a similar EF and FS to that of WT mice, while older Het mice displayed a significantly reduced EF and FS compared with WT mice, which indicated that aging advanced the reduction of cardiac function induced by MAVS deficiency. In addition, left ventricular (LV) dilation was observed in MAVS^-/-^ mice, as assessed by the expanded LV volume at the end of the systolic/diastolic stage (LVVs/LVVd) and enlarged LV internal diameter in the systolic/diastolic stage (LVIDs/LVIDd) (**Figure S1**). The cross-sectional view of hearts at the papillary level also supported cardiac dilation in mice lacking MAVS (**Figure 2D**). However, though the LV mass was increased in mice without MAVS, the interventricular septum at the end of the systolic/diastolic stage (IVSs/IVSd) and the left ventricular posterior wall at the end of the systolic/diastolic stage (LVPWs/LVPWd) were not different between WT and MAVS^-/-^ mice, although young Het mice displayed a slight increase trend, which may be result of compensatory mechanism during the early stage (**Figure S1**). These data indicate MAVS ablation could induce cardiac contractibility decrease and cardiac dilation, but cardiac hypertrophy was not developed. Therefore, MAVS plays an important role in maintaining normal cardiac function and that MAVS lack results in progressive cardiac dysfunction with aging.

### MAVS Depletion Disrupted Cardiac Energy Metabolism by Disturbing Lipid Metabolism

**Figure 3:**
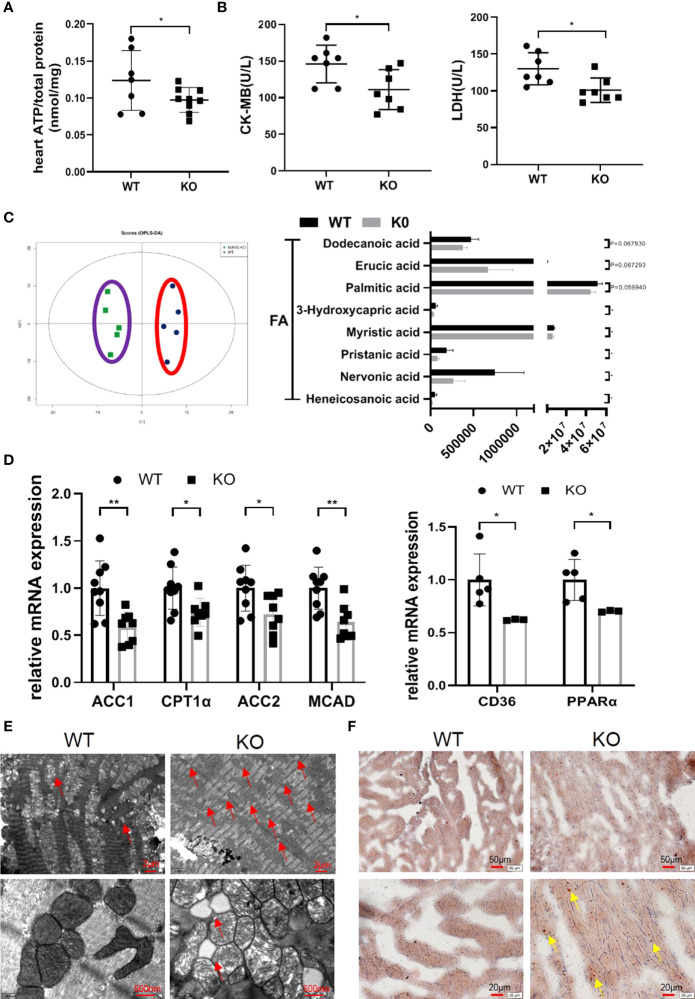
MAVS deficiency resulted in energy metabolism disorder by disturbing lipid metabolism. **(A)**. The ATP content in the hearts of MAVS^-/-^ mice was lower than that in WT mice (n: WT=7, knockout [KO]=9). **(B)**. Serum CK-MB and LDH levels in WT and MAVS^-/-^ mice (n: WT=7, KO=7). **(C)**. OPLS-DA analysis based on non-targeted metabolomics was conducted in the hearts of WT and MAVS^-/-^ mice (n: WT=5, KO=5). The levels of some medium- and long-chain fatty acids were decreased in MAVS^-/-^ mice (n: WT=5, KO=5). **(D)**. RT-qPCR analysis showed downregulation of fatty acid metabolism related genes (ACC1/2, CPT1α, MCAD) in the hearts of MAVS^-/-^ mice (n: WT=9, KO=8), reduced expression of CD36 and PPARα (n: WT=5, KO=3). **(E)**. Representative TEM images of hearts from WT and MAVS^-/-^ mice (Upper panel, scale bar: 2 µm; Lower panel, scale bar: 500 nm, red arrow: lipid droplets). **(F)**. Representative Oil Red O-stained images of hearts from WT and MAVS^-/-^ mice (Upper panel, scale bar: 50 µm; Lower panel, scale bar: 20 µm, yellow arrow: lipid droplets). The quantitative results are shown as the means ± SEM (*P < 0.05 and **P < 0.01).

Next, we examined how MAVS deficiency advanced cardiac dysfunction. Considering the importance of adenosine triphosphate (ATP) in myocardial contraction and relaxation, we wondered whether MAVS depletion affects ATP production in the heart in an unstressed state. The level of ATP in fresh heart tissues was decreased in MAVS^-/-^ mice in accordance with the loss of cardiac function (**Figure 3A**). Since CK-MB and LDH are abundant in the heart and play crucial roles in ATP production, we next detected the concentrations of CK-MB and LDH in serum of MAVS^-/-^ mice and found that both were remarkably lower than those in WT mice (**Figure 3B**).

To further investigate the influence of MAVS deletion on cardiac energy metabolism, untargeted metabonomics of hearts from WT and MAVS^-/-^ mice were performed. Orthogonal partial least squares discriminant analysis score plots showed that WT and MAVS^-/-^ mice were completely separated (positive ion mode: R2X=0.423, R2Y=0.969, Q2 = 0.00144), suggesting dysregulated metabolism in MAVS^-/-^ mice (**Figure 3C**). Hierarchical cluster analysis of the metabolomic data is shown in **Figure S3A**. Compared to WT mice, MAVS^-/-^ mice showed reduced levels of several medium- and long-chain fatty acids (heneicosanoic acid, nervonic acid, pristanic acid, myristic acid, 3-hydroxycapric acid, palmitic acid, erucic acid, and dodecanoic acid) (**Figure 3C**). Fatty acids are an important energy resource for the heart, and their reduction may contribute to the incapacity of myocardial contraction. We also observed downregulated levels of multiple phosphatidylcholines (PCs) including 1,2-dioleoyl-sn-glycero-3-phosphatidylcholine, 1,2-dipalmitoyl-sn-glycero-3-phosphorylcholine [PC (16:0/16:0)], N-docosanoyl-4-sphingenyl-1-o-phosphorylcholine, 1-stearoyl-sn-glycerol 3-phosphocholine, and 1-stearoyl-2-oleoyl-sn-glycerol 3-phosphocholine (SOPC), whereas the levels of PC catabolites (1-palmitoylglycerol and glycerophosphocholine) were increased in the hearts of MAVS^-/-^ mice (**Figure S2B**). Except for PCs, phosphatidylethanolamides (PEs) and sphingomyelins (SMs) were also reduced in MAVS^-/-^ mice versus WT mice (**Figure S2C**). PC, PE, and SM are essential components of the cell membrane, indicating that MAVS deletion might increase cell membrane decomposition or decrease cellular turnover, fueling limited cardiomyocyte growth. Inspired by the metabolite variations, we hypothesized that MAVS deficiency affected fatty acid metabolism. Therefore, quantitative reverse transcription polymerase chain reaction (RT-qPCR) was used to assess the levels of some genes involved in fatty acid metabolism. As shown in **Figure 3D**, at the mRNA level, genes involved in fatty acid synthesis [acetyl-CoA carboxylase 1 (ACC1)], uptake [cluster of differentiation 36 (CD36)], transport [carnitine palmitoyltransferase 1α (CPT1α)], and oxidation [peroxisome proliferator-activated receptor α (PPARα), acetyl-CoA carboxylase 2 (ACC2), and medium-chain acyl-CoA dehydrogenase (MCAD)] were downregulated (**Figure 3D**). These data suggest that the general decrease in fatty acid metabolism resulting from MAVS deficiency may contribute to reduced energy generation in the heart. Interestingly, increased lipid accumulation was observed in MAVS^-/-^ mice, as indicated by more lipid droplets in the transmission electron microscope (TEM) images of the hearts (**Figure 3E**). Consistently, Oil red O (ORO) staining also showed more droplets in MAVS^-/-^ mice (**Figure 3F**). Lipid accumulation may be indicative of excessive lipid uptake or lipid utilization disorder, therefore, whether loss of MAVS impairs fatty acid oxidation (FAO) requires further investigation. We may conclude that MAVS depletion affected energy metabolism by disturbing lipid metabolism, accelerating the decrease of cardiac function.

### MAVS Deletion Aggravated Mitochondrial Dysfunction by Inducing Mitochondrial Oxidative Stress and Impaired Mitophagy

**Figure 4:**
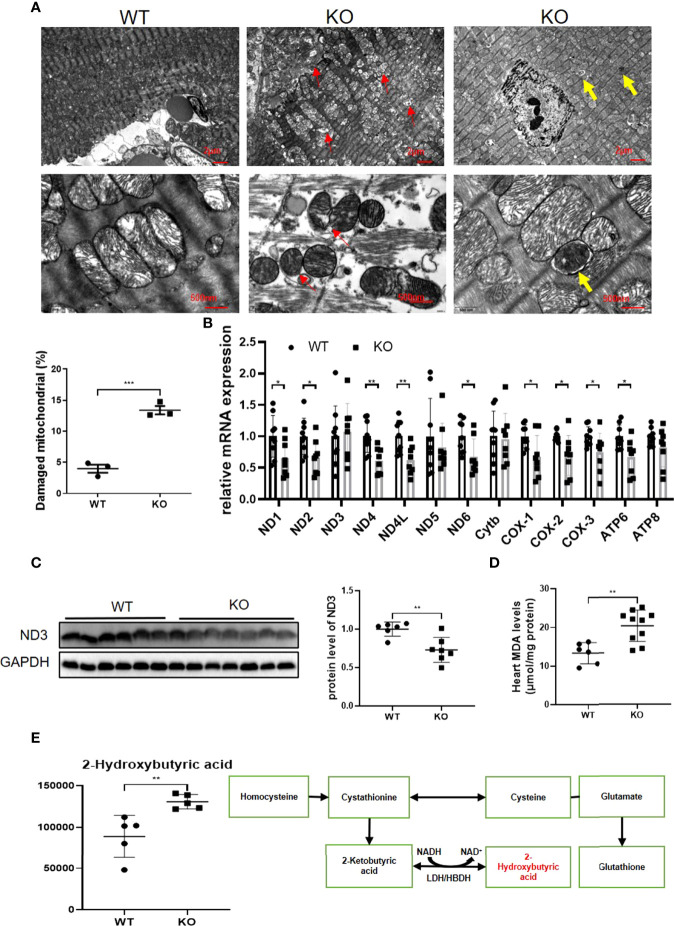
MAVS deletion resulted in mitochondrial dysfunction by inducing oxidative stress and impaired mitophagy. **(A)**. Representative TEM images of hearts showed breaks in myocardial fibers, mitochondrial disorganization, and mitophagy (Upper panel, scale bar: 2 µm; lower panel, scale bar: 500 nm; red circle: myocardial fiber rupture; red arrow: damaged mitochondria; yellow arrow: autophagosomes containing mitochondrion). Quantification of damaged mitochondria in the hearts of MAVS-/- mice by TEM images (n: WT=3, KO=3). **(B)**, RT-qPCR analysis of mitochondrial genes in the hearts of WT and MAVS^-/-^ mice (n: WT=9, KO=8). **(C)**. Western blotting analysis showed reduced expression of mitochondrial electron transfer chain protein ND3 in MAVS-/- mice. Densitometry analysis of the western blots of ND3 (n: WT=6, KO=7). **(D)**. Increased level of MDA was detected in MAVS^-/-^ mice hearts (n: WT=6, KO=10). **(E)**. The level of 2-hydroxybutyric acid was markedly increased in the hearts of MAVS^-/-^ mice (n: WT=5, KO=5). 2-Hydroxybutyric acid is a byproduct in the synthesis of glutathione in response to oxidative stress. The quantitative results are shown as the means ± SEM (*P < 0.05, **P < 0.01 and ***P < 0.001).

Since mitochondrial dysfunction is tightly associated with cardiac dysfunction, we next examined whether mitochondria were damaged in MAVS^-/-^ mice. Using TEM, we observed ballooned mitochondria with disrupted or disappeared ridges in the hearts of MAVS^-/-^ mice versus WT mice (**Figure 4A**). Consistent with the destroyed mitochondrial structure, the expression of some mitochondrial genes was reduced at the mRNA and protein levels in MAVS^-/-^ mice, suggesting a decrease in mitochondrial oxidative phosphorylation capacity (**Figures 4B, C**).

Since oxidative stress could lead to mitochondrial damage, we next investigated whether oxidative stress existed in MAVS^-/-^ mice. Malondialdehyde (MDA) levels were markedly higher in the hearts of MAVS^-/-^ mice than in WT mice, indicating an oxidative stress state in MAVS^-/-^ mice (**Figure 4D**). Further analysis of the metabonomics data revealed that 2-hydroxybutyric acid was strikingly higher in MAVS^-/-^ mice than in WT mice (**Figure 4E**). 2-Hydroxybutyric acid is an organic acid derived from 2-ketobutyric acid, a byproduct of glutathione synthesis in response to oxidative stress. Therefore, the high concentration of 2-hydroxybutyric acid may be a result of excessive substrate 2-ketobutyric acid resulting from oxidative stress. Thus, MAVS depletion may aggravate mitochondrial damage by inducing oxidative stress. Conversely, mitochondrial damage could lead to increased ROS production, forming a vicious cycle.

Notably, autophagosomes were observed in MAVS^-/-^ mice, but not in WT mice when we scrutinized the TEM images of the hearts (**Figure 4A**). The observed mitochondrial autophagosomes suggested that a lack of MAVS might activate mitophagy to diminish damaged mitochondria resulting from MAVS deficiency to maintain mitochondrial homeostasis. However, impaired mitophagy activation may exacerbate mitochondrial dysfunction.

### MAVS Knockout by CRISPR/Cas9 in H9C2 Cells Resulted in Mitochondrial Dysfunction

**Figure 5:**
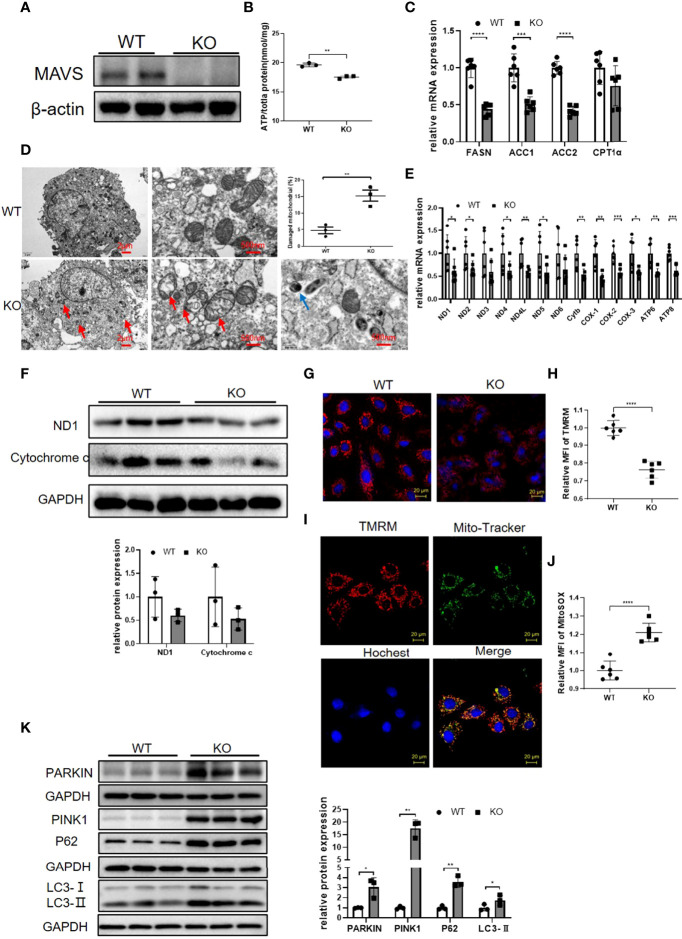
Loss of MAVS resulted in mitochondrial dysfunction in H9C2 cells. **(A)**. Representative western blotting verified the deletion of MAVS by CRISPR/Cas9 in H9C2 cells. **(B)**. ATP production was reduced in H9C2-/- cells. **(C)**. RT-qPCR analysis of some genes (FASN, ACC1, ACC2, CPT1α) involved in lipid metabolism in H9C2-/- cells (n=6) **(D)**. Representative TEM images of damaged mitochondria and autophagosomes in H9C2-/- cells, (Left panel, scale bar: 2 µm; Right panel, scale bar: 500 nm; red arrow: damaged mitochondria; blue arrow: autophagosomes). Quantification of damaged mitochondria in H9C2-/- cells by TEM images (n=3). **(E)**. RT-qPCR analysis for mitochondrial genes in control and H9C2-/- cells (n=6). **(F)**. Western blotting analysis of mitochondrial electron transfer chain proteins ND1 and Cytochrome C in H9C2-/- cells. Densitometry analysis of the western blots of ND1 and Cytochrome C (n=3). **(G)**. Representative fluorescence images of tetramethylrhodamine, methyl ester (TMRM) in H9C2-/- cells (original magnification, 400×; scale bar: 20 µm). **(H)**, Quantitative analysis of MFI of TMRM by flow cytometry (n=6). **(I)**. Representative fluorescence images from confocal microscope confirmed the staining signals of MMP was from mitochondria. TMRM (red); Mito-tracker staining (green); Hoechst (blue); merge image (yellow) (original magnification, 400×; scale bar: 20 µm). **(J)**. Quantitative analysis of MFI of MitoSOX by flow cytometry in H9C2 cells (n=6). **(K)**. Western blotting analysis of mitophagy marker proteins Parkin, PINK1, LC3-II, and P62. Densitometry analysis of the western blots of above proteins (n=3). The quantitative results are shown as the means ± SEM (*P < 0.05, **P < 0.01, ***P<0.001 and ****P < 0.0001).

To illustrate the MAVS function in myocytes, we generated MAVS-deficient H9C2 (H9C2-/-) using the CRISPR/Cas9 method and western blotting verified the successful deletion of MAVS in H9C2 cells (**Figure 5A**). Since MAVS-/- mice displayed impaired energy metabolism, we firstly investigated whether loss of MAVS could result in similar phenomenon in myocytes. In agreement with the *in vivo* results, the ATP level was significantly reduced in H9C2-/- cells versus with naïve cells (**Figure 5B**). Paralleled with reduced ATP content, a general downregulation of fatty acid metabolism was detected in H9C2-/- cells, which was also consistent with that of MAVS-/- mice (**Figure 5C**).Given that MAVS is located at the OMM and may play a crucial role in maintaining mitochondrial function, we next evaluated mitochondrial structural changes by TEM. Visually, the TEM revealed mitochondrial injury in H9C2-/- cells. As shown in **Figure 5D**, an increase in circularity, blurred or disappeared mitochondrial ridges, mitochondrial vacuolization, and incomplete disruption of the mitochondrial outer membrane were observed in H9C2-/- cells. In addition, we observed downregulation of mitochondrial genes involved in the electron transfer chain at mRNA and protein levels in H9C2-/- cells, which was in line with that observed in MAVS^-/-^ mice (**Figures 5E, F**). Since mitochondrial damage is usually followed by a decrease in mitochondrial membrane potential (MMP), we measured the MMP level of H9C2 cells. Con-focal images and Flow cytometry analysis of control and H9C2-/- cells both demonstrated reduced MMP levels (**Figures 5G, H**). Co-location of TMRM with Mito-Tracker detected by laser confocal microscope (LSCM) also confirmed that the staining signals of MMP was from mitochondria instead of other organelles (**Figure 5I**). In addition, MitoSOX™ Red mitochondrial superoxide indicator staining revealed increased mitochondrial ROS in H9C2-/- cells, a finding that was coordinated to data from MAVS^-/-^ mice (**Figure 5J**). The relative oxidative stress state in H9C2-/- cells may contribute to mitochondrial damage. Interestingly, we also observed mitochondrial autophagosomes in H9C2-/- cells, that were not detected in control cells (**Figure 5D**). Then, we further examined the expression of some mitophagy marker proteins by western blotting analysis (**Figure 5K**). Results showed that Parkin and PINK1 were remarkably upregulated in H9C2-/- cells accompanied by an increased level of LC3-II, however, adaptor protein P62 was also increased, suggesting that MAVS loss impaired the mitophagy flux. Therefore, MAVS deficiency may contribute to mitochondrial damage by inducing mitochondrial ROS generation and abnormal mitophagy.

### MAVS Deletion Led to Similar Cardiac Dysfunction in Female Mice

**Figure 6:**
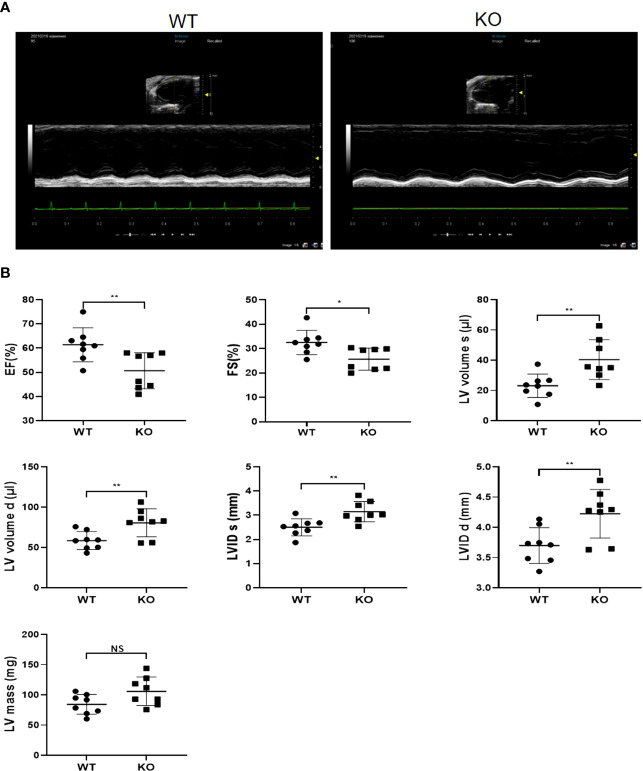
MAVS deletion leads to left ventricular dilation and systolic function decrease in female mice. **(A)**, Representative short axis M-mode echocardiographic images of female WT and MAVS^-/-^ mice at the age of 6 months. **(B)**, EF, FS, LVVs/d, LVIDs/d, and LV mass of mice in WT and MAVS^-/-^ mice. The quantitative results were shown as the means ± SEM (NS not significant, *p < 0.05 and **p < 0.01).

To determine whether MAVS deficiency cause a sex-based difference, we also evaluated the cardiac function of female MAVS^-/-^ mice by echocardiography. As shown in **Figure 6**, 24-week-old female MAVS^-/-^ mice exhibited reduced cardiac contractility and ventricular dilation, which was similarly to that of male mice. However, LV mass was not significantly different between WT and MAVS^-/-^ mice, with only a slight trend toward an increase. Therefore, MAVS depletion did not cause obvious differences in heart function between male and female mice.

### MAVS Deficiency did not Affect Cardiac Development

**Figure 7:**
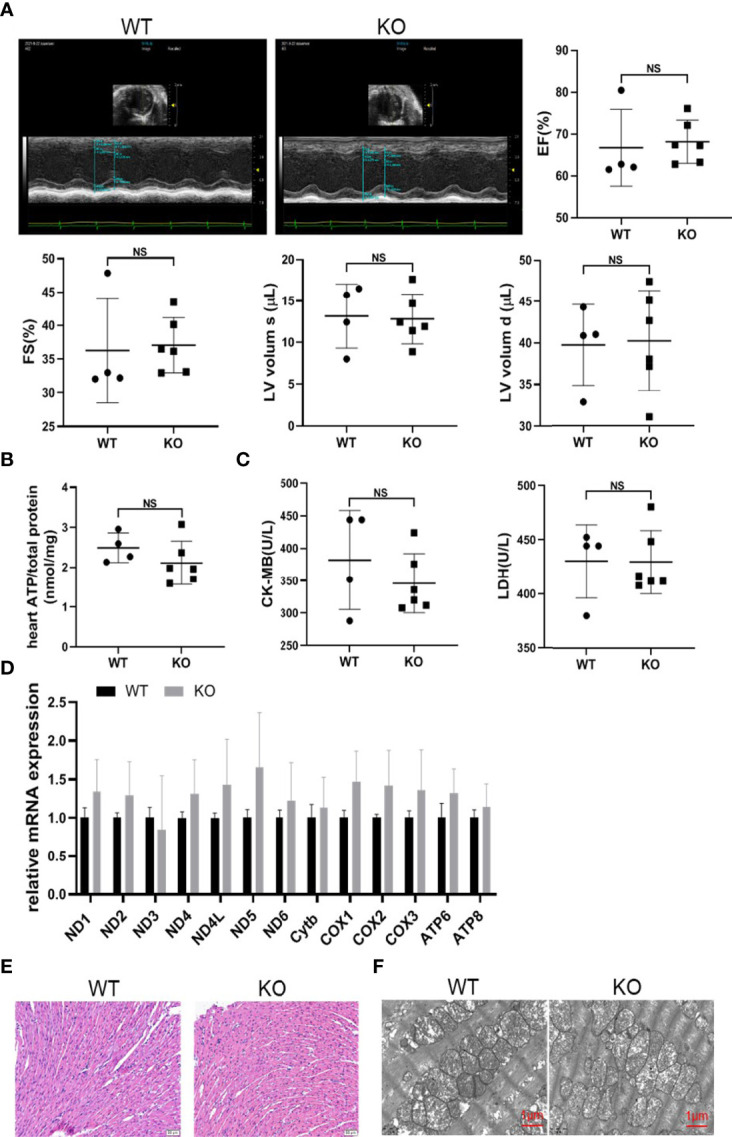
MAVS deficiency did not affect cardiac development. **(A)**. Representative short-axis M-mode echocardiographic images of WT and MAVS^-/-^ mice at the age of 2-3 weeks. EF, FS, and LVVs/d did not differ between WT and MAVS^-/-^ mice. **(B)**. The ATP content in the hearts of mice did not differ between WT and MAVS^-/-^ mice (n: WT=4, KO=6). **(C)**. Serum CK-MB and LDH levels in mice (n: WT=4, KO=6). **(D)**, RT-qPCR analysis of mitochondrial genes in the hearts of WT and MAVS^-/-^ mice (n: WT=4, KO=6). **(E)**. Representative HE staining images of hearts from WT and MAVS^-/-^ mice revealed no morphologic changes of cardiomyocytes (scale bar: 20 µm). **(F)**. Representative TEM images of hearts showed no intergroup difference in mitochondrial morphology (scale bar: 500 nm). The quantitative results are shown as the means ± SEM. (NS not significant).

Since MAVS^-/-^ mice gradually developed a decline in cardiac function and became worse with aging, we must exclude the possibility that this phenotype was a result of congenital cardiac hypoplasia induced by MAVS deficiency. Therefore, we detected the cardiac function of MAVS^-/-^ mice aged 2-3 weeks regardless of sex on echocardiography. The results showed no difference in cardiac function supported by parameters including EF, FS, LVV, LVID, and LV mass between WT and MAVS^-/-^ mice (**Figures 7A** and **S3**). The ATP content of hearts from MAVS^-/-^ mice was also analyzed, and a lack of MAVS seemed to have no significant effects on ATP production in young mice (**Figure 7B**). Next, we determined the serum CK-MB and LDH levels and found no decline in mice without MAVS, supporting the ATP results (**Figure 7C**). Consistently, mitochondrial function did not differ between groups, although there was an increasing trend of mitochondrial gene expression resulting from MAVS loss, which might be transient compensation (**Figure 7D**). Hematoxylin-eosin (HE) staining and TEM data showed no significant morphologic variations in cardiomyocytes and mitochondria in WT and MAVS^-/-^ mice (**Figures 7E, F**). Thus, these data suggest that MAVS deletion might not influence cardiomyocyte development and could not lead to mitochondrial structure and function changes in young mice. However, as mice mature, their energy demands increase gradually and mitochondria without MAVS fail to produce enough ATP, therefore, the hearts weaken.

## Discussion

Here we described for the first time that MAVS deletion led to a gradual decline in cardiac systolic function and cardiac dilation in mice. This might be partly explained by impaired energy metabolism induced by mitochondrial dysfunction, oxidative stress, and abnormal mitophagy due to loss of MAVS function. MAVS deficiency consistently resulted in mitochondrial damage, impaired mitophagy, and oxidative stress in H9C2 cells.

MAVS is well recognized as a key signaling protein downstream of viral RNA sensors RIG-1 and MDA5 during viral infection (9, 14). MAVS also participates in EAE, multiple sclerosis, and SLE (10, 11, 15). Besides the regulation of immune response, a recent study demonstrated that MAVS coordinated with NOD1/RIP2 to promote cardiac remodeling following pressure overload by regulating the NF-κB and mitogen activated protein kinase (MAPK)-GATA4/P300 pathways (13). Similar, we also found MAVS was upregulated in the hearts of Angiotensin II (Ang II)-treated mice, which might be a result of transcription of mitogenic factors induced by Ang II. Differing from the upregulation of MAVS in TAC or Ang II-induced cardiac hypertrophy, MAVS was downregulated in LPS, IR or 5/6Nx-induced non-hypertrophic cardiac dysfunction. Here, in our study, in the unstressed state, MAVS^-/-^ mice displayed reduced cardiac function characterized by decreased myocardial contractility and ventricular enlargement compared to WT mice. Additionally, this spontaneous phenotype did not differ between the sexes but was associated with age. Therefore, MAVS may perform differently in various pathological and physiological conditions. The increased expression of MAVS in TAC-induced cardiac hypertrophy may be explained by compensated mitochondrial biogenesis to meet the requirements for maintaining a certain cardiac output. On the contrary, loss of MAVS resulting from damaged mitochondria or other insults under LPS, IR or 5/6Nx stimuli may lead to the mitochondrial injury and cardiac dysfunction.

Energy metabolism dysfunction is a major cause of cardiac dysfunction. CK-MB, which serves as a mobile energy pool, is crucial for ATP generation since it transfers the high-energy phosphate bond of creatinine phosphate to ADP. Previous studies have shown that CK-MB activity was markedly reduced in the hearts of patients with heart failure (HF) as well as the failing hearts of mice induced by transverse aortic constriction and coronary artery ligation (16, 17). In addition, mice deficient in CK-MB could develop congestive HF (18). Similarly, we observed reduced CK-MB levels in the serum of MAVS^-/-^ mice. Moreover, the decreased LDH level in MAVS^-/-^ mice supported a previous study showing reduced LDH activity in failing patient hearts (19). To some extent, the lower CK-MB and LDH levels could contribute to the reduced ATP content in MAVS^-/-^ mice.

Lipids are the major energy resource in the heart, and 50-70% of ATP is generated from fatty acid oxidation (20). Mounting evidence has illustrated the existence of lipid metabolism disorders in cardiac dysfunction. Multiple phospholipids, including PCs, LysoPCs, LysoPEs, and SMs, were reduced in the serum of patients with HF, which was related to more severe clinical symptoms or complications (21–23). Similarly, our metabonomics data showed a general reduction of PCs, PEs, and SMs in the hearts of MAVS^-/-^ mice. A previous study also found a low abundance of medium- and long-chain acyl carnitine in the hearts of patients with advanced HF (24). Similarly, we observed decreased levels of several medium- and long-chain fatty acids as well as the downregulation of some genes involved in fatty acid metabolism. Thus, lipid metabolism disturbances accelerated the exacerbation of cardiac function in MAVS^-/-^ mice. In addition, MAVS^-/-^ mice exhibited more lipid accumulation than WT mice, consistent with a recent study indicating that MAVS ablation aggravated diet-induced lipid accumulation in the liver by upregulating the relevant lipogenic pathway (25). Lipid aggregation was detected in the hearts of HF patients (especially those with obesity or diabetes) as well as in the hearts of acute ischemia mice (26, 27). In addition, more lipid droplets accumulated in myocytes as cardiac function decreased further by (28). Lipid accumulation can induce endoplasmic reticulum stress and mitoROS production, resulting in myocyte apoptosis and cardiac dysfunction (29). Therefore, the decrease of cardiac function in MAVS^-/-^ mice may be explained by the disturbance of lipid metabolism and the resulting lipotoxicity induced by MAVS depletion. However, the underlying mechanism by which MAVS deletion causes lipid metabolism disorder remains unclear and requires further research. Prohibitins (PHBs) are essential for lipid metabolism and mitochondrial homeostasis (30, 31). It was reported PHB2 deficiency could lead to impaired cardiac FAO, mitochondrial dysfunction and heart failure (32). Considering the location of PHB2 in the inner membrane of mitochondria (IMM), we may speculate MAVS deletion might disturb lipid metabolism by affecting some IMM proteins such as PHB2. Notably, a recent study demonstrated the link between MAVS and PHBs. Caseinolytic peptidase B protein homolog (CLPB), a mitochondrial intermembrane space (IMS) protein, together with other mitochondrial proteins including A-kinase-anchoring protein 1(AKAP1) and ATPase family AAA-domain containing protein 3 A (ATAD3A), served as a bridge connecting PHB complex with MAVS (33). Besides, SLC25A12, an aspartate-glutamate transporter localized in the IMM, could also interact with MAVS (34). These findings suggest that MAVS deficiency might disrupted its interaction with some IMM or IMS proteins involved in lipid maturation, leading to lipid metabolism disturbance.

Enormous studies have reported that mitochondrial dysfunction is a major contributor to the decline of cardiac function and that the restoration or improvement of mitochondrial function can prevent or delay the outcome of HF (4, 7, 35, 36). In our experiment, MAVS^-/-^ mice exhibited prominent mitochondrial structure damage and functional decline as supported by TEM and RT-qPCR results. A similar phenomenon was observed in H9C2 cells lacking MAVS. Due to the location of MAVS in the OMM, we next examined how MAVS affects the mitochondria. We found that some genes involved in the electron transfer chain were significantly downregulated at the mRNA level in MAVS^-/-^ mice, indicating a reduced mitochondrial respiration capacity. However, in recent studies, MAVS knockdown significantly increased the oxygen consumption rate, spare respiratory capacity, and ATP-linked respiration in isolated neonatal cardiomyocytes and mouse embryonic fibroblasts (10, 13). Silencing MAVS also enhanced mitochondrial complex I activity in cardiomyocytes (13). From this perspective, MAVS seems to be an inhibitor of mitochondrial function, which is contrary to our data showing decreased ATP and MMP levels in H9C2-/- cells. However, in agreement with our results, Fu et al. suggested that MAVS deletion exacerbated mitochondrial dysfunction by reducing mitochondrial respiratory capacity (25). We may explain that partial inhibition MAVS might cause compensatory promotion of mitochondrial function *via* unknown mechanisms to sustain normal cell respiration and energy metabolism. Unfortunately, complete or persistent loss of MAVS damaged mitochondrial structure and function, and therefore mitochondrial activity was reduced. Additionally, the metabolic profiles of neonatal cardiomyocytes differ from those of adult cardiomyocytes. Therefore, further investigations are warranted to uncover the detailed mechanism by which MAVS modulates mitochondrial function.

Mitochondrial dysfunction can lead to oxidative stress, conversely ROS can aggravate mitochondrial injury, forming a vicious cycle. Oxidative stress can accelerate cardiac insufficiency through multiple mechanisms, including cardiomyocyte electrophysiology disruption, calcium overload, and further mitochondrial dysfunction (37). We found that MAVS^-/-^ mice had higher MDA levels and displayed a relative oxidative stress state. In addition, higher level of mitoROS was observed in MAVS-deficient H9C2 cells. In the absence of viral infection, chemically induced oxidative stress can induce MAVS oligomerization and the activation of downstream signaling pathways (10). Conversely, except for sensing viral infection signaling, MAVS could also sense cellular stress and activate the antioxidant response by activating NF-κB signaling pathway. Thus, the lack of MAVS weakened the cellular resistance to various stimuli and caused a comparative oxidative stress state. More importantly, MAVS loss-induced mitochondrial damage further increased mitoROS generation.

Mitophagy is an important mechanism that maintains mitochondrial quality and quantity. Mounting evidence has indicated that mitophagy protects the heart, while its impairment contributes to cardiac dysfunction. PINK1 was downregulated in the hearts of end-stage HF patients, and PINK1^-/-^ mice also showed age-dependent cardiac hypertrophy and ultimately developed HF (38). Loss of Parkin, another mitophagy participant, predisposed mice to myocardial infarction (MI), although it did not affect cardiac function physiologically in WT versus Parkin^-/-^ mice (39). Interestingly, mice lack of Fundc1 (a mitophagy receptor) exhibited aggravated cardiac injury and cardiac dysfunction after acute MI than mice with heterozygous deletion of autophagy-related gene Beclin1, indicating mitophagy may play more prominent role in cardiac protection than general autophagy (40). Though autophagy or mitophagy is usually activated in response to various stress, we observed increased mitochondrial autophagosomes in the hearts of MAVS^-/-^ mice as well as in H9C2-/- cells without any stress. Besides, we also observed upregulated Parkin, PINK1 and LC3-II in H9C2-/- cells. However, adaptor protein P62 was also increased, suggesting that MAVS loss could impair the mitophagy flux. Recent studies have described the role of MAVS in maintaining mitochondrial homeostasis by mediating autophagy. During viral infection, the polyubiquitin translocation of MAVS induced by RNF34 (a cytosolic E3 ubiquitin ligase) could be recognized by NDP52 (ubiquitin receptor), resulting in the degradation of impaired mitochondria by autophagy (41). In addition, the spontaneous aggregation of MAVS could induce ROS production and Bcl-2 interacting protein 3 like (BNIP3L, also named NIX)-mediated autophagic clearance of MAVS aggregates in response to viral infection (42). More importantly, MAVS can directly interact with LC3 *via* its LC3 interacting region (LIR) motif ‘Y(9)xxI (12)’ in the CARD domain, inducing autophagy activation (43, 44). These studies indicate the important role of MAVS in the regulation of autophagy and support our findings in this study.

## Conclusions

Our results demonstrate that MAVS is downregulated in hearts with non-hypertrophic cardiac dysfunction and reduced MAVS contributes to the decline of cardiac function. Energy metabolism disturbance and mitochondrial dysfunction are involved in the MAVS deficiency-mediated cardiac dysfunction progression. This study broadens our knowledge of the pathogenesis of cardiac insufficiency and may provide therapeutic potential by targeting MAVS.

## Data Availability Statement

The raw data supporting the conclusions of this article will be made available by the authors, without undue reservation.

## Ethics Statement

The animal study was reviewed and approved by Institutional Animal Care and Use Committee of Nanjing Medical University. Written informed consent was obtained from the owners for the participation of their animals in this study.

## Author Contributions

ZJ, WX and YZ designed the experiments. QW and WX analyzed data and wrote the manuscript. QW, WX, ZS, SC, XL, MW, YL, JY, and WZ performed the experiments. SH, AZ contributed to technical advices, and all authors reviewed the manuscript.

## Funding

This study was supported by the National Natural Science Foundation of China (81873599, 82070760, 81830020, 82070701, 81974084, 82090022, 82170754, 82100779, 81800652 and 81600352); The National Key Research and Development Program (2019YFA0802700); the Jiangsu High-Level Innovation and Entrepreneurship Talent Introduction Program (2019SCJH001); Natural Science Foundation of Jiangsu Province (BK20200153); and the Nanjing National Commission on Health and Family Planning (ZKX19042).

## Conflict of Interest

The authors declare that the research was conducted in the absence of any commercial or financial relationships that could be construed as a potential conflict of interest.

## Publisher’s Note

All claims expressed in this article are solely those of the authors and do not necessarily represent those of their affiliated organizations, or those of the publisher, the editors and the reviewers. Any product that may be evaluated in this article, or claim that may be made by its manufacturer, is not guaranteed or endorsed by the publisher.
