# Supplementary material for: Reduced Immunity Regulator MAVS Contributes to Non-Hypertrophic Cardiac Dysfunction by Disturbing Energy Metabolism and Mitochondrial Homeostasis
Source: Front Immunol. 2022 Jul 1;13:919038. doi: 10.3389/fimmu.2022.919038 (PMC9283757; doi:10.3389/fimmu.2022.919038)
Supplement: Supplementary file 1 [file DataSheet_1.pdf]

Supplementary Material

Supplementary Figures and Figure legends

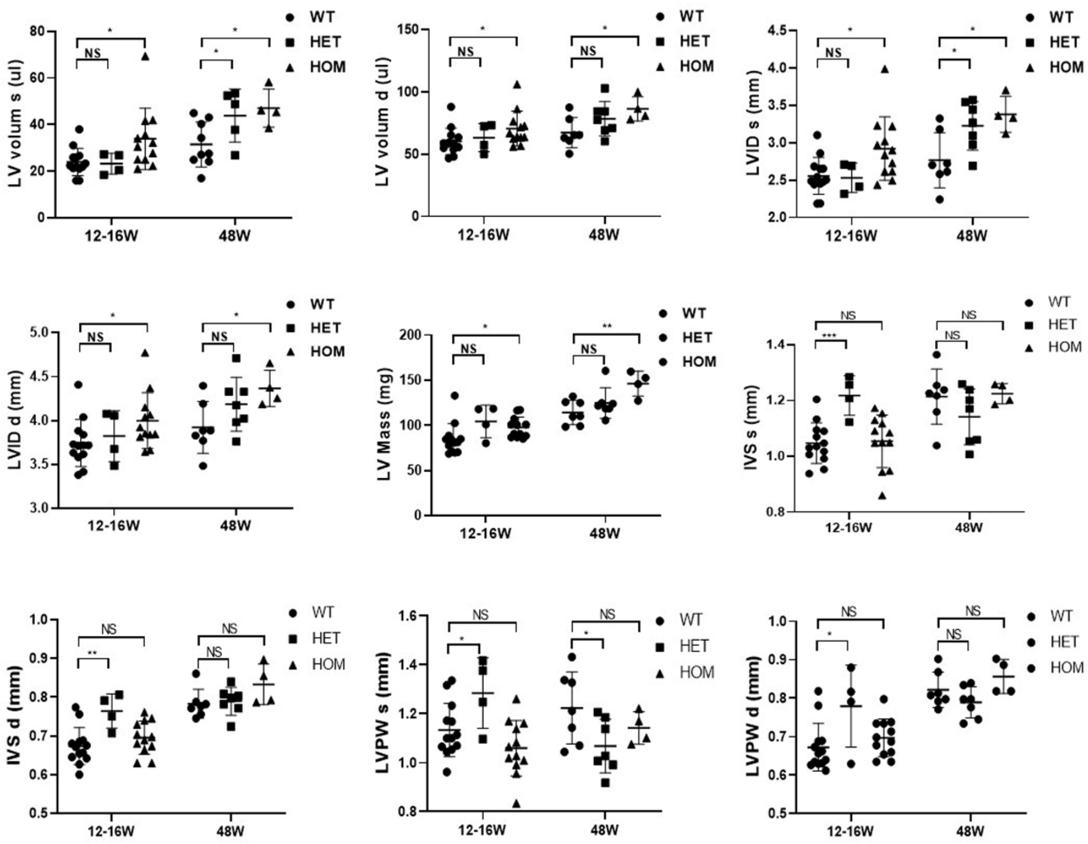

Figure S1. MAVS deficiency led to left ventricle dilation without cardiac hypertrophy. LVIDs/d, Left ventricular internal diameter in systolic/diastolic. LVVs/d, Left ventricular volume at the end of systolic/diastolic stage. LV mass, Left ventricular LV mass. IVSs/d, Interventricular septum at the end of systolic/diastolic stage. LVPWs/d, Left ventricular posterior wall at the end of the systolic/diastolic stage. The quantitative results were shown as the means ±SEM (NS not significant, * $p<0.05$ and ** $p<0.01$).

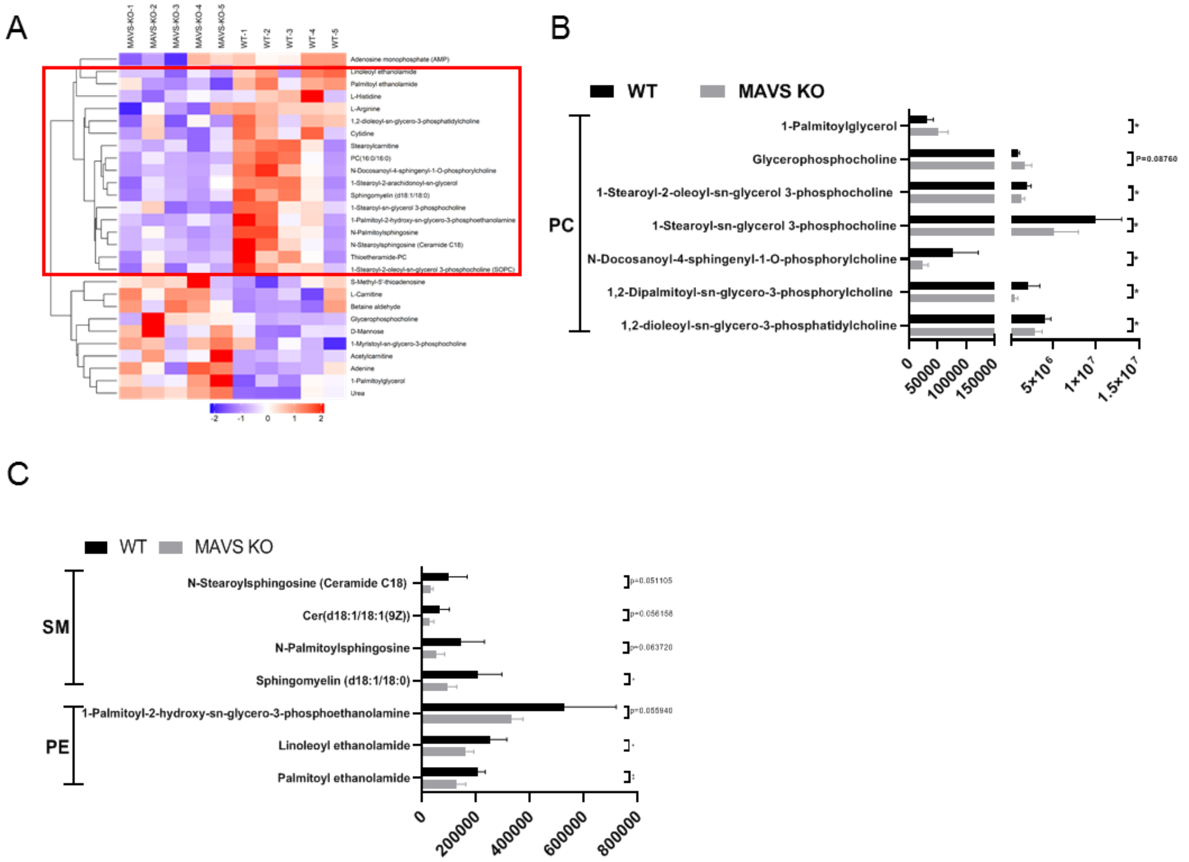

Figure S2. MAVS loss led to lipid metabolism disorder. A, Hierarchical cluster analysis was used to characterize the metabolites that differed between WT and MAVS$^{-/-}$ mice. B, The levels of phosphatidylcholines (PCs) were reduced in MAVS$^{-/-}$ mice. C, The levels of sphingomyelins (SMs) and phosphatidylethnolamines (PEs) were lower in MAVS-/- mice (n: WT=5, KO=5). The quantitative results were shown as the means ± SEM (* p<0.05 and ** p<0.01).

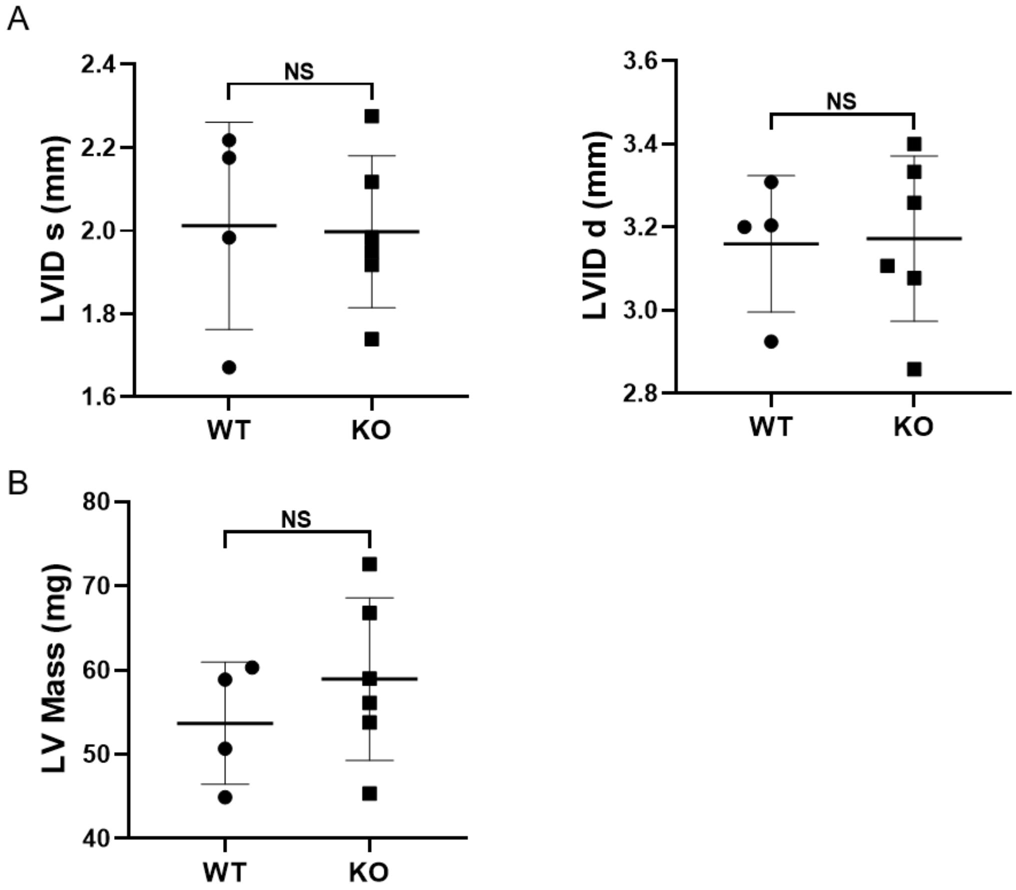

Figure S3. MAVS deficiency did not affect cardiac development. A, LVIDs/d did not differ between WT and KO mice aged 2-3 weeks. B, LV mass was not increased in MAVS$^{-/-}$ mice. The quantitative results were shown as the means ± SEM (NS, not significant).
